# Dietary Fiber Pectin Ameliorates Experimental Colitis in a Neutral Sugar Side Chain-Dependent Manner

**DOI:** 10.3389/fimmu.2019.02979

**Published:** 2019-12-19

**Authors:** Keita Ishisono, Toshiyuki Mano, Tomio Yabe, Kohji Kitaguchi

**Affiliations:** ^1^United Graduate School of Agricultural Science, Gifu University, Gifu, Japan; ^2^Graduate School of Natural Science and Technology, Gifu University, Gifu, Japan; ^3^Department of Applied Life Science, Faculty of Applied Biological Sciences, Gifu University, Gifu, Japan; ^4^Center for Highly Advanced Integration of Nano and Life Sciences (G-CHAIN), Gifu University, Gifu, Japan

**Keywords:** colitis, IL-6, inflammation, macrophage, pectin

## Abstract

Dietary fiber, with intake of soluble fibers in particular, has been reported to lower the risk for developing inflammatory bowel diseases (IBD). This is at least partly attributable to the fermentation of dietary fiber by the colonic microbiota to produce short chain fatty acids. Pectin, a widely consumed soluble fiber, is known to exert a protective effect in murine models of IBD, but the underlying mechanism remains elusive. Apart from having a prebiotic effect, it has been suggested that pectin direct influences host cells by modulating the inflammatory response in a manner dependent on its neutral sugar side chains. Here we examined the effect of the side chain content of pectin on the pathogenesis of experimental colitis in mice. Male C57BL/6 mice were fed a pectin-free diet, or a diet supplemented with characteristically high (5% orange pectin) or low (5% citrus pectin) side chain content for 10–14 days, and then administered 2,4,6-trinitrobenzene sulfonic acid or dextran sulfate sodium to induce colitis. We found that the clinical symptoms and tissue damage in the colon were ameliorated in mice that were pre-fed with orange pectin, but not in those pre-fed with citrus pectin. Although the population of CD4^+^Foxp^+^ regulatory T cells and CD4^+^RORγt^+^ inflammatory T cells in the colon were comparable between citrus and orange pectin-fed mice, colonic interleukin (IL)-1β and IL-6 levels in orange pectin-fed mice were significantly decreased. The fecal concentration of propionic acid in orange pectin-fed mice was slightly but significantly higher than that in control and citrus pectin-fed mice but the cecal concentration of propionic acid after the induction of TNBS colitis was comparable between orange and citrus pectin-fed mice. Furthermore, the protective effect of orange pectin against colitis was observed even in mice treated with antibiotics. IL-6 production from RAW264.7 cells stimulated with the toll-like receptor agonist Pam3CSK4 or lipopolysaccharide was suppressed by pre-treatment with orange pectin *in vitro*. Taken together, these results suggest that the side chains of pectin not only augment prebiotic effects but also directly regulate IL-6 production from intestinal host cells in a microbiota-independent fashion to attenuate colitis.

## Introduction

Inflammatory bowel diseases (IBD) consisting mainly of ulcerative colitis and Crohn's disease are idiopathic inflammatory disorders of the gastrointestinal tract. Epidemiological studies indicate that over 1.2 million people in North America and 2 million people in Europe have suffered from IBD (1–4), with a prevalence that exceeds 0.3% in North America, Oceania, and Europe. In addition, the prevalence of IBD is rising not only in developed countries but also in newly industrialized countries in Africa, Asia, and South America (5). Although the precise etiology of IBD remains unclear, they are thought to be the result of an aberrant mucosal immune response to the commensal microflora in the gut in combination with susceptible genotypes (6). In addition to genetic factors, dietary habits are also associated with the development of IBD.

A high-fiber diet including soluble fibers in particular has been reported to protect against the development of IBD (7, 8), and a prospective cohort study has suggested that high, long-term intake of dietary fiber lowers the risk of Crohn's disease by 40% (9). Furthermore, this protective effect against IBD is thought to be at least partly attributable to the fermentation of dietary fiber by the colonic microbiota, which then produce short chain fatty acids (SCFAs) including acetic acid, propionic acid, and butyric acid. SCFAs become the main energy source of colonic epithelial cells and inhibit immune abnormality via the G protein-coupled receptor (GPR) and epigenetic pathways, which result in alleviation of colonic inflammation (10–13). In addition to the prebiotic effect, there are particular carbohydrate molecules in dietary fiber that directly interact with host cells to modulate immune responses. Pectin, a water-soluble dietary fiber, has a known prebiotic effect (14, 15), but several studies have indicated that pectin directly regulates intestinal inflammation apart from its prebiotic effect (16, 17). Popov et al. showed that a single oral dose of pectin exhibited a protective effect against acetic acid-induced colitis (18). In a cell-based assay, pectin treatment downregulated toll-like receptor (TLR) 4 signaling and inhibited interleukin (IL)-1β production in human peripheral blood mononuclear cells (19). However, the microbiota-independent pathway by which pectin exerts its anti-inflammatory effect is poorly understood.

Pectin belongs to a family of complex polysaccharides and is a major component of the middle lamella in terrestrial plants. Pectin mainly consists of a linear homo-polymer of α-1,4-linked-d-galacturonic acid residues (principal chain), which is partly esterified with methyl and acetyl groups. The degree of methyl esterification (DM) of pectin differs between plant species and affects fermentation in the cecum, SCFA production, and anti-inflammatory activity (17, 20). In addition, the principal chain of pectin is covalently linked to the rhamnogalacturonan (RG)-I and RG-II (21), both of which construct the side chain regions in the pectin molecule. RG-I mainly consists of neutral sugar chains including arabinan, galactan, and arabinogalactan, while RG-II consists of galactose, arabinose, rhamnose, apiose, aceric acid, 3-deoxy-lyxo-2-heptulosaric acid, and 3-deoxy-manno-2-octulosonic acid. Although the side chain content of pectin also varies between plant species (22), there is no clear evidence demonstrating a protective effect of the side chain content *per se* against gastrointestinal inflammation.

Our previous study demonstrated that pectin suppressed inflammatory cytokine production in Peyer's patch CD11c^+^ cells and attenuated systemic inflammation in mice (16). Furthermore, the anti-inflammatory effect required the neutral sugar side chain of pectin. Accordingly, we hypothesized that the side chain of pectin exerts its protective effect against colitis by modulating the production of colonic SCFAs and/or direct interacting with intestinal host cells. In the present study, we investigated the role of pectin side chains in a mouse model of experimental colitis using dietary pectins with high and low side chain content, and explored a possible mechanism for its anti-colitis effect.

## Materials and Methods

### Reagents

Citrus pectin (derived from lemon and lime peels) and orange pectin were kindly provided by CP Kelco ApS (Lille Skensved, Denmark). Dextran sulfate sodium (DSS) and 2,4,6-trinitrobenzoic sulfonic acid (TNBS) and were obtained from Wako Pure Chemical Industries (Osaka, Japan). Lipopolysaccharide (LPS, *Escherichia coli* O111: B4) and Pam3CSK4 were purchased from Sigma-Aldrich (St. Louis, MO, USA) and Tocris Bioscience (Bristol, UK), respectively.

### Mice

Male C57BL/6 mice were purchased from CLEA Japan (Shizuoka, Japan), and were housed in individual cages with free access to water and food. Mice were fed either the AIN-93G diet (pectin-free control) or a modified AIN-93G diet supplemented with 5% citrus pectin or orange pectin for the entirety of the experiments, and were maintained at a constant temperature of 23°C ± 1°C with a daily 12-h light/12-h dark cycle. All experiments were performed on 7–8-week-old mice.

### TNBS-Induced Colitis

TNBS colitis was induced according to a method described by Wirtz et al. (23) with slight modifications. Between 7 and 8 days before TNBS sensitization, mice were started on pectin-supplemented diets, and then were sensitized with 1% TNBS in mixture of acetone and olive oil on the back shoulder. Fourteen days after initiation of the pectin feeding, mice were lightly anesthetized by inhalation of isoflurane (Wako), followed by intrarectal administration of 100 μL 2.5% TNBS dissolved in 50% ethanol using a 3.5-Fr catheter equipped with a 1-mL syringe. The catheter tip was inserted 4 cm proximal to the anal verge and mice were kept in a head-down position for 90 s after intrarectal injection to ensure distribution of the TNBS solution within the colon lumen. Body weight and food intake were monitored daily. Colon and cecum were collected at days 2 or 3 after the TNBS injection for further analysis.

### Histopathology

Colon samples were fixed in 10% neutral-buffered formalin for 24 h. After fixation, the samples were embedded in paraffin, cut into 4-μm sections, and stained with hematoxylin and eosin. Histological signs of colitis were evaluated in a blinded fashion using a previously reported scoring system (24) based on inflammatory cell infiltration and tissue damage as follows. Inflammatory cell infiltration scoring: 0 = presence of occasional inflammatory cells in the lamina propria; 1 = increased numbers of inflammatory cells in the lamina propria; 2 = confluence of inflammatory cells extending into the submucosa; and 3 = transmural extension of the infiltrate. Tissue damage scoring: 0 = no mucosal damage; 1 = lymphoepithelial lesions; 2 = surface mucosal erosion or focal ulceration: and 3 = extensive mucosal damage and extension into deeper structures of the bowel wall.

### Preparation of Lamina Propria Cells

Colonic lamina propria cells were prepared according to a method described by Couter et al. (25) with slight modifications. The colon was collected 2 days after TNBS challenge and was inverted using curved forceps. The tissue was incubated in RPMI-1640 medium (Nissui Pharmaceutical, Tokyo, Japan) containing 5 mM EDTA, 33 μM dithiothreitol (Wako), 1.6% fetal bovine serum (FBS) and 100 units/mL penicillin-streptomycin for 15 min at 37°C with stirring. After the incubation, the tissue was minced and then incubated in RPMI-1640 containing 0.8 mg/mL collagenase (Wako), 0.04 mg/mL DNase I (Worthington Biochemical, Lakewood, NJ, USA), 1.2% FBS and 100 units/mL penicillin-streptomycin for 40 min at 37°C with stirring to create a single cell suspension. The cells were resuspended in 3 mL 100% Percoll (GE Healthcare, Little Chalfont, England) and then covered with 3 mL 40% Percoll. Percoll gradient separation was performed by centrifugation at 800 × g for 20 min at 4°C. Cells in the intermediate layer were subjected to flow cytometry.

### Flow Cytometry

Colonic lamina propria cells were incubated with anti-CD16/32 antibody (clone 2.4G2, Tonbo Biosciences, San Diego, CA, USA) to block Fc receptors and then stained with fluorescein isothiocyanate-conjugated anti-CD4 antibody (clone RM4-5, Biolegend, San Diego, CA, USA). Subsequently, the cells were fixed and permeabilized with true-nuclear transcriptional factor buffer set (Biolegend) then stained with phycoerythrin (PE)-conjugated anti-Foxp3 antibody (clone MF-14, Biolegend), PE-conjugated anti-RORγt antibody (clone B2D, eBioscience, San Diego, CA, USA) or Alexa fluor 647-conjugated anti-T-bet antibody (clone 4B10, Biolegend). After washing the cells with phosphate-buffered saline containing 0.5% bovine serum albumin, the fluorescence intensity was measured by flow cytometry (FACSCalibur; BD Biosciences, San Jose, CA, USA). Data were analyzed using CellQuest software (BD Biosciences).

### Measurement of SCFAs

Fecal samples and cecum contents were collected 14 and 17 days after starting the pectin-supplemented diets, respectively. Each sample (20 mg) was suspended at a ratio of 1:50 (w/v) in MilliQ water, and disrupted using zirconia beads. After centrifugation at 10,000 × g for 10 min, SCFAs in the supernatant were measured using a YMC pack FA kit (YMC, Kyoto, Japan) and high-pressure liquid chromatography (HPLC) system (PU-2089 Plus, JASCO, Tokyo, Japan). The column was maintained at 50°C with a mobile phase consisting of acetonitrile-methanol-water (30:16:54 v/v/v, pH 4–5 adjusted by 0.1 M HCl) delivered at a flow rate of 0.5 mL/minute. Labeled SCFAs were detected at a wavelength of 400 nm using a UV/Visible HPLC detector (UV-2075 Plus, JASCO).

### Antibiotic Treatment

Antibiotics were given according to a method described by Chinen et al. (26) with slight modifications. In brief, mice were orally administered meropenem trihydrate (50 mg/kg/day, Wako) and vancomycin hydrochloride (50 mg/kg/day, Wako) 3 days before pectin feeding, and then continued to being given the antibiotics for 19 days.

### Enzyme-Linked Immunosorbent Assay (ELISA)

Concentrations of inflammatory cytokines in colon tissue were measured by commercial ELISA kit according to the manufacturer's instructions. Mouse IL-1β, IL-6, interferon (IFN)- γ, and tumor necrosis factor (TNF)-α levels were determined using Duoset ELISA (R&D Systems, Minneapolis, MN, USA) and IL-17A levels was measured with IL-17A ELISA kit (Biolegend). The absorbance was measured at 450 nm with a microplate reader (Tecan, Mannedorf, Switzerland). The concentrations of colonic cytokines were normalized to the total intestinal protein concentration as determined by a bicinchoninic acid assay kit (ThermoFisher scientific, Waltham, MA, USA).

### DSS-Induced Colitis

Mice were fed pectin-supplemented diets for 10 days before being given 3% DSS in drinking water for 8 days. Body weight, disease activity index (DAI), and food intake were monitored daily. The DAI was calculated by the summation of previously reported criteria (27) based on the following: weight loss percentage (0 = none; 1 = 1 to 5%; 2 = 5 to 10%; 3 = 10 to 20%; 4 = > 20%); fecal bleeding (0 = no bleeding; 1 = a few blood-tinged stools; 2 = some bleeding; 3 = gross bleeding; 4 = blood filling the whole colon); and stool consistency (0 = normal stool; 1 = slightly loose stool; 2 = loose stools; 3 = watery stool; 4 = severe diarrhea). Colon samples were collected 8 days after DSS administration for further analysis.

### Enzymatic Digestion of Pectin

Polysaccharides derived from side chain of pectin were prepared as described previously (16). Briefly, aliquots of 1 mg/mL pectin dissolved in acetic acid buffer were mixed with 10 μL of pectinase solution (Pectinex ultra SPL; Sigma-Aldrich) and incubated at 50°C for 24 h. Subsequently, the reaction solution was dialyzed against deionized water for 3 days using Spectra/Por Dialysis Membranes (molecular weight cut-off, 6,000–8,000 Da; Spectrum Laboratories, Rancho Dominguez, CA, USA) and lyophilized. The complete degradation of pectin was confirmed by size exclusion chromatography.

### Cell Culture

The murine macrophage cell line RAW264.7 was obtained from American Type Culture Collection (Manassas, VA, USA) and cultured in Dulbecco's modified Eagle medium (Nissui) supplemented with 10% FBS and 100 units/mL penicillin-streptomycin at 37°C under 5% CO_2_. RAW264.7 cells were seeded at a density of 3.0 × 10^5^ cells/mL in 96-well culture plates and incubated with 250 μg/mL pectin or 50 μg/mL side chain-derived polysaccharides for 24 hours. After pectin treatment, cells were stimulated with 1 μg/mL LPS or Pam3CSK4, and the supernatants were collected 24 hours after the stimulation for the measurement of IL-6 concentration by ELISA as described above.

### Statistics

All results are expressed as means ± standard error of the mean. Data were analyzed by one-way analysis of variance followed by the Tukey–Kramer test or Dunnett's test. Differences between means were considered significant at *P* < 0.05.

## Results

### Orange Pectin, but Not Citrus Pectin, Ameliorates TNBS-Induced Colitis

**Figure 1:**
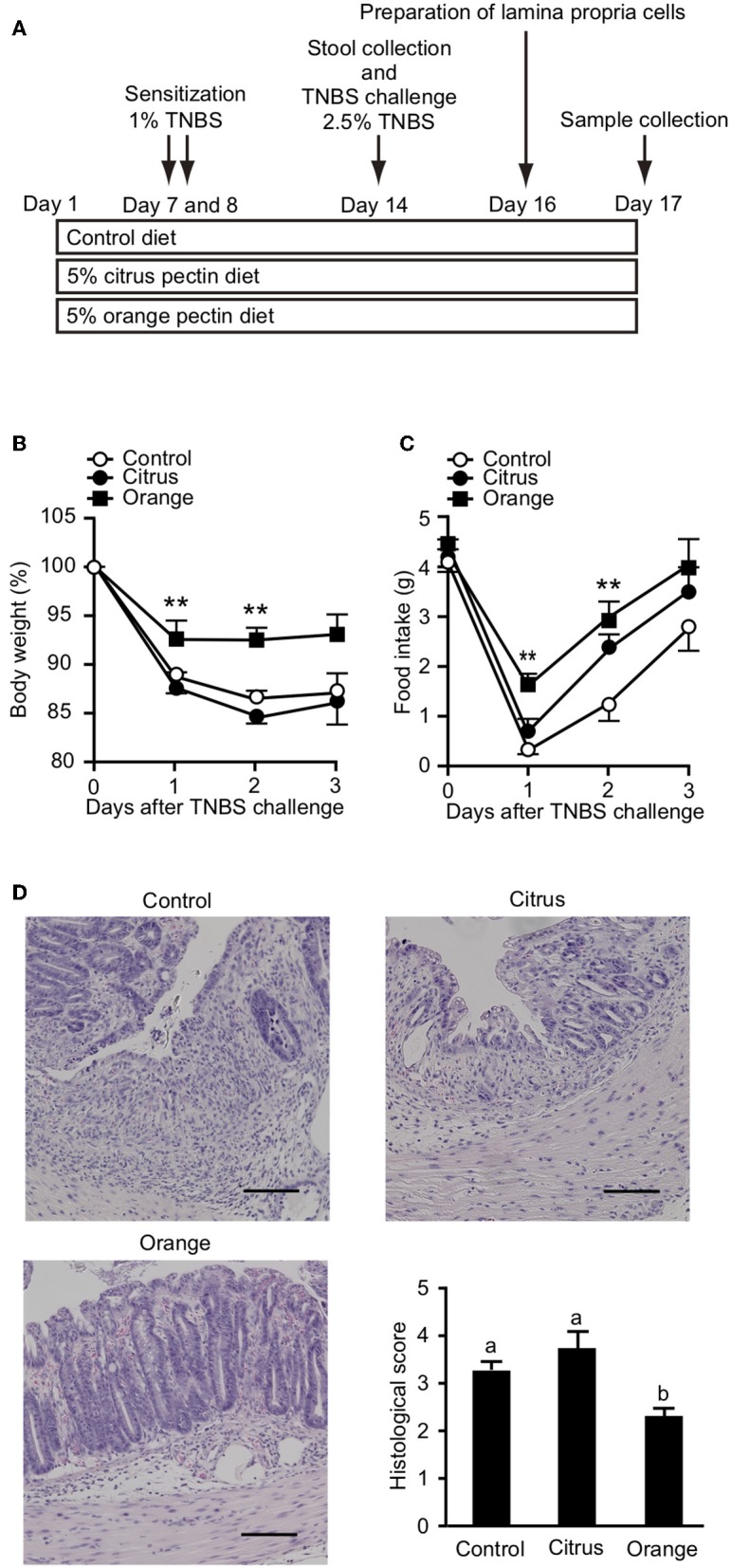
Effects of pectin feeding on trinitrobenzoic sulfonic acid (TNBS)-induced colitis in mice. **(A)** Experimental design and time-course of treatment. Change in **(B)** body weight and **(C)** food intake after TNBS challenge. **(D)** Histological score 3 days after TNBS challenge. Scale bars: 100 μm. Values are presented as means ± standard error of the mean (*n* = 8–9). ^**^P < 0.01. Values not sharing a common letter (a or b) were significantly different (*P* < 0.05).

It has been previously reported that orange pectin contains 1.9 times more arabinose and 2.5 times more galactose compared with citrus pectin; however, the side chain lengths, average molecular weight, and DM are comparable between the two (28). Accordingly, orange pectin has a higher number of neutral sugar side chains than citrus pectin (Supplementary Figure 1). To investigate preventive effects of the side chain content of pectin on TNBS-induced colitis, mice were fed a diet supplemented with orange or citrus pectin for 14 days, and then challenged with TNBS (Figure 1A). Control mice that were fed a pectin-free diet lost more than 10% body weight and decreased their food intake by approximately 3 g on day 2 following TNBS challenge (Figures 1B,C). Compared with control mice, orange pectin-fed mice had less weight loss and a milder decrease in food intake in response to TNBS challenge (Figures 1B,C). There were trends toward reduced weight loss and decrease in food intake in orange pectin-fed mice even 3 days after TNBS challenge. Moreover, histopathological analysis showed low levels of epithelial damage and inflammatory cell infiltration in orange pectin-fed mice (Figure 1D). Conversely, amelioration of TNBS-induced colitis was not observed in citrus pectin-fed mice (Figures 1B–D). Collectively, these results indicated that pectin ameliorated TNBS-induced colitis in a side chain-dependent manner.

### Protection Against TNBS Colitis by Orange Pectin Is Not Due to Regulation of Colonic T Cell Differentiation

**Figure 2:**
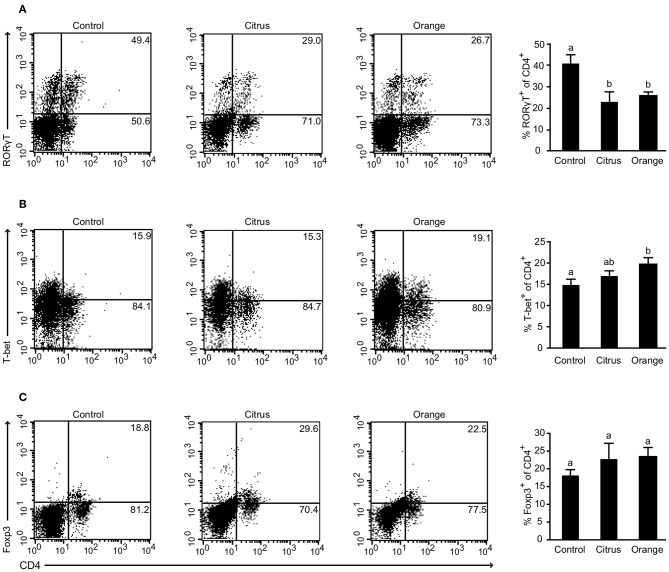
Effect of pectin feeding on colonic T cell differentiation in mice with trinitrobenzoic sulfonic acid (TNBS)-induced colitis. Colonic lamina propria cells analyzed by flow cytometry 2 days after TNBS challenge showing percentages of **(A)** CD4+RORγt+ cells; **(B)** CD4+T-bet+ cells; and **(C)** CD4+Foxp3+ cells. Values are presented as means ± standard error of the mean (*n* = 4–5). Values not sharing a common letter (a or b) were significantly different (*P* < 0.05).

To gain further insight into the mechanism by which orange pectin ameliorated TNBS colitis in a side chain-dependent manner, we attempted to identify the type of cells involved. Previous studies demonstrated that several helper T cell subsets infiltrate the murine colon in relation to the pathogenesis and restoration of TNBS-induced colitis (29). In particular, Th17 and Th1 cells, defined by their expression of RORγt and T-bet, respectively, are believed to aggravate TNBS-induced colitis by producing distinct cytokines including IL-17 and INF-γ (30, 31). Flow cytometric analysis showed that the frequency of CD4^+^RORγt^+^ cells (Th17) was significantly decreased in pectin-fed mice compared with control mice; however, there was no significant difference between citrus and orange pectin (Figure 2A), suggesting that the protective effect of orange pectin against TNBS colitis was not attributable to suppression of Th17 differentiation. Interestingly, the frequency of CD4^+^T-bet^+^ (Th1) cells was significantly increased only in orange pectin-fed mice (Figure 2B). In contrast to Th17 and Th1, regulatory T cells (Treg), which can be defined by expression of Foxp3, are capable of suppressing the activation of Th17 and Th1 cells through the production of IL-10 and transforming growth factor-β in mouse colitis models (29). Accordingly, we investigated whether orange pectin augments colonic Treg differentiation. The frequency of colonic CD4^+^Foxp3^+^ cells (Treg) was not changed by feeding with either orange or citrus pectin (Figure 2C). These results indicated that pectin feeding affected the differentiation of colonic Th1 cells in these mice.

### Orange Pectin Downregulates Colonic Inflammatory Cytokines in TNBS Colitis

**Figure 3:**
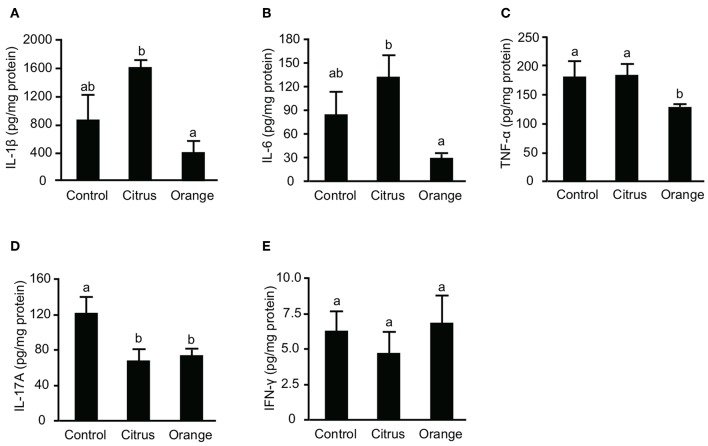
Effects of pectin feeding on colonic inflammatory cytokine production in mice with trinitrobenzoic sulfonic acid (TNBS)-induced colitis. Levels of **(A)** interleukin (IL)-1β, **(B)** IL-6, **(C)** tumor necrosis factor (TNF)-α, **(D)** IL-17A, and **(E)** interferon (IFN)-γ levels 3 days after TNBS administration. Values are presented as means ± standard error of the mean (*n* = 8–9). Values not sharing a common letter (a or b) were significantly different (*P* < 0.05).

**Figure 4:**
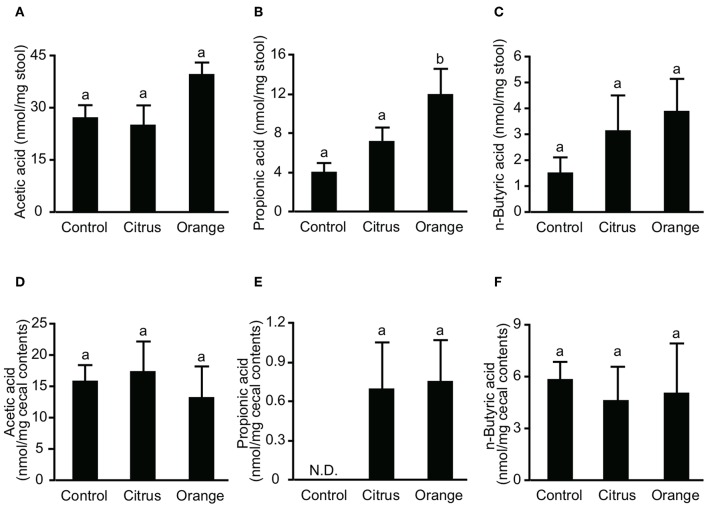
Effect of pectin feeding on the production of three intestinal short-chain fatty acids in mice. Stools and cecum contents were collected at 14 and 17 days after pectin feeding, respectively, and used to determine the concentrations of **(A)** fecal acetic acid, **(B)** fecal propionic acid, **(C)** fecal butyric acid, **(D)** cecal acetic acid, **(E)** cecal propionic acid, and **(F)** cecal butyric acid. Values are presented as means ± standard error of the mean (*n* = 5). Values not sharing a common letter (a or b) were significantly different (*P* < 0.05).

Previous studies reported that not only effector T cells but also myeloid cells, including macrophages and dendritic cells, are committed to the pathogenesis of TNBS colitis (32, 33) and that their inflammatory cytokines are principal mediators of mucosal inflammation. To confirm that pectin dampens the production of colonic cytokines, we evaluated the production of IL-1β, IL-6, and TNF-α in the colon. The levels of colonic IL-1β, IL-6, and TNF-α in orange pectin-fed mice were significantly lower than those in citrus pectin-fed mice and there was also a trend toward lower IL-1β and IL-6 levels compared with control mice (Figures 3A–C). As colonic Th17 and Th1 populations were changed by pectin feeding (Figure 3), we also measured concentration of prototypical Th cytokines, *i.e*., IL-17A for Th17 and INF-γ for Th1. Consistent with the number of colonic Th17 (Figure 3A), IL-17A level was significantly decreased in both citrus and orange pectin-fed mice (Figure 4D). On the other hand, colonic IFN-γ level was not changed with or without pectin feeding (Figure 4E). These results suggested that the protective effect of orange pectin against TNBS-induced colitis was mediated by the suppression of inflammatory cytokine production in the colon.

### Orange Pectin Temporary Increases Fecal Propionic Acid, but Has No Effect After Colitis Induction

SCFAs are major effector metabolites for the prebiotic effect and are involved in helper T cell differentiation and cytokine production. A previous report demonstrated that butyric acid augmented Treg differentiation by inhibiting histone deacetylase activity (11), and decreased the expression of IL-6 from macrophages and dendritic cells via GPR-109a (34). To investigate whether orange pectin could augment SCFA production by the colonic microbiota, we measured the concentrations of fecal SCFAs in pectin-fed mice. Consistent with the number of colonic Treg (Figure 2C), the concentrations of acetic acid and butyric acid in stool samples were comparable with or without 14 days of pectin feeding (Figures 4A,C). On the other hand, the fecal concentration of propionic acid in orange pectin-fed mice was significantly higher than that of control and citrus pectin-fed mice (Figure 4B). However, the concentrations of cecum SCFAs including propionic acid were comparable between orange and citrus pectin fed-mice 3 days after TNBS challenge (Figures 4D–F). These results suggested that orange pectin had a marginal effect on augmentation of propionic acid production only in a healthy colon without colitis.

### Orange Pectin Ameliorates TNBS Colitis, Even in Antibiotic-Treated Mice

**Figure 5:**
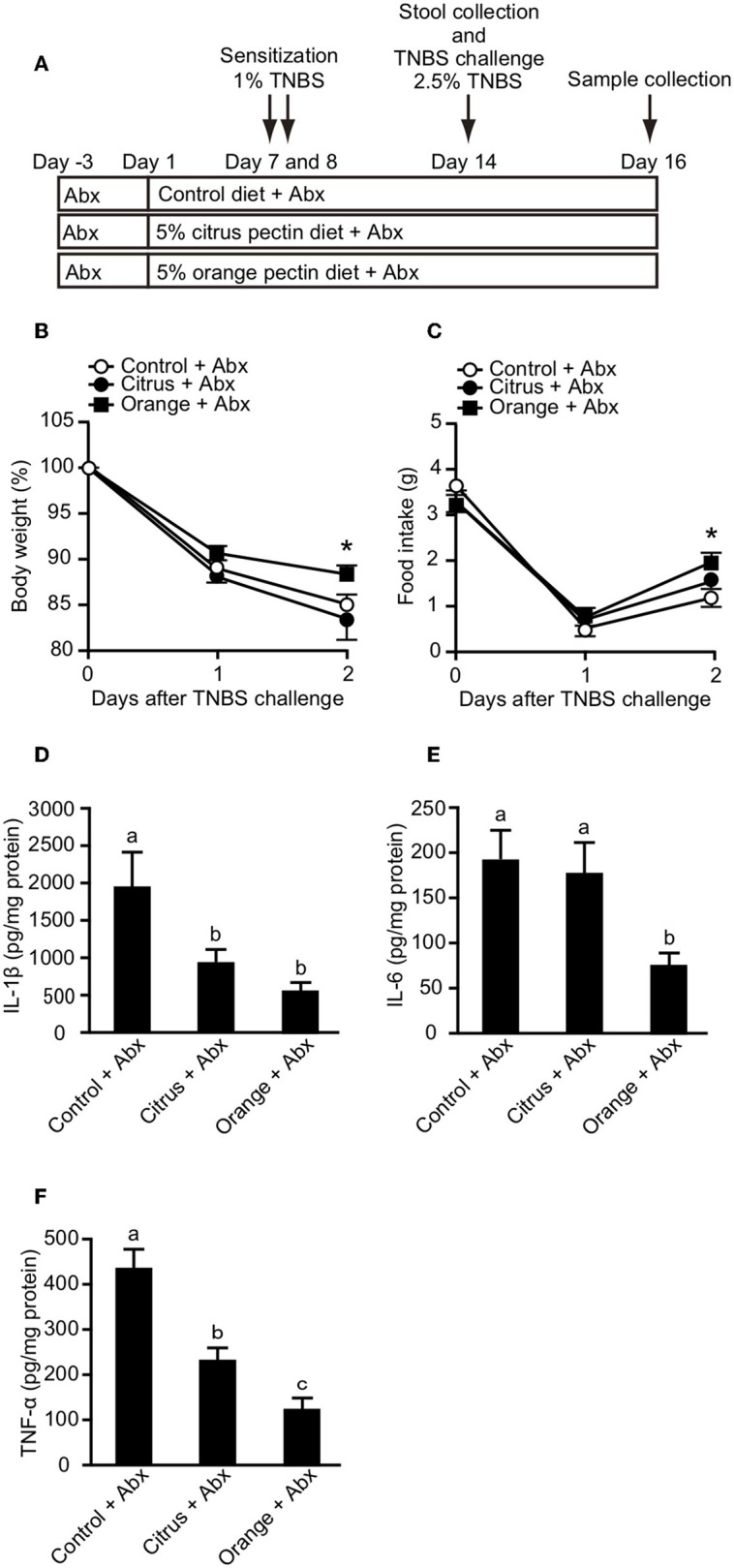
Effects of pectin feeding on trinitrobenzoic sulfonic acid (TNBS)-induced colitis in mice pre-treated with two antibiotics (Abx). **(A)** Experimental design and time-course of treatment. Changes in **(B)** body weight and **(C)** food intake after TNBS challenge. Colonic **(D)** interleukin (IL)-1β, **(E)** IL-6, and **(F)** tumor necrosis factor (TNF)-α levels 3 days after TNBS challenge. Values are presented as means ± standard error of the mean (*n* = 9–12). Values not sharing a common letter (a—c) were significantly different (*P* < 0.05). ^*^P < 0.05.

Propionic acid has been reported to regulate cytokine production via GPR-41 (35) and GPR-43 (36). Because orange pectin temporary increased the fecal concentration of propionic acid (Figure 4B), we investigated whether the protective effect of orange pectin against colitis was related to intestinal fermentation by the colonic microbiota. To this end, fecal SCFA levels were determined following a 19-day course of two antibiotics (b). The levels of all three fecal SCFAs were clearly decreased following antibiotic treatment, but were comparable between the control and pectin-fed mice (Supplementary Figure 2). Moreover, antibiotic treatment also reduced total genomic copy number of bacterial 16S rRNA in feces to <1/100 (Supplementary Figure 3, Supplementary Information). It has been previously reported that antibiotic treatment or germ-free bleeding of murine model of IBD is associated with markedly less severe intestinal inflammation because of loss of commensal bacterial antigen to induce colitis (37, 38), but TNBS-haptenized autologous antigen also induces both innate and adaptive immune responses in TNBS colitis even without microflora (23, 39). When mice pre-treated with antibiotics were challenged with TNBS, the control and citrus pectin-fed mice exhibited progressive weight loss and decreased food intake, while orange pectin-fed mice showed significant attenuation of weight loss and decreased food intake compared with control mice 2 days after TNBS challenge (Figures 5B,C). Furthermore, the levels of colonic IL-1β, IL-6, and TNF-α in orange pectin-fed mice were significantly decreased compared with control mice (Figures 5D–F). Citrus pectin-fed mice also showed significant decreases in the production of cytokines IL-1β and TNF-α (Figures 5D,F), but not IL-6 (Figure 5E). Nevertheless, feeding with citrus pectin did not diminish the effects of TNBS on weight loss and reduction in food intake (Figures 5B,C). Several studies have suggested that neutralization of IL-6 attenuates murine colitis (40, 41), while Terabe et al. went further, demonstrating that neutralization of the IL-6 receptor specifically had stronger therapeutic efficacy than neutralization of TNF-α (42). These results indicated that orange pectin could attenuate TNBS-induced colitis via downregulation of IL-6 production, independent of its prebiotic effect.

### Orange Pectin Directly Suppresses IL-6 Production Triggered by TLR1/2 and TLR4 Stimulation in Macrophages

**Figure 6:**
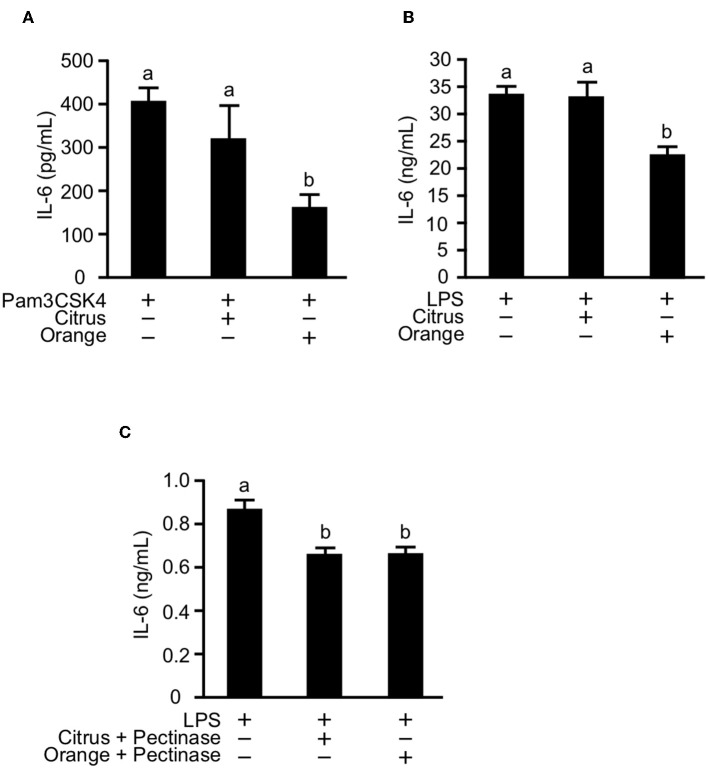
Effect of pectin pre-treatment on interleukin (IL)-6 production in RAW264.7 macrophages. Cells were pre-treated with 250 μg/mL citrus or orange pectin for 24 h and stimulated with 1 μg/mL **(A)** Pam3CSK4 or **(B)** LPS. **(C)** Cells were pre-treated with 50 μg/mL polysaccharides derived from side chain of citrus or orange pectin for 24 hours and stimulated with 1 μg/mL LPS. The IL-6 concentration in the supernatant was determined 24 h after toll-like receptor stimulation. Values are presented as means ± standard error of the mean of three independent experiments. Values not sharing a common letter (a or b) were significantly different (*P* < 0.05).

Previous studies reported that the activation of TLR2 and TLR4 expression on macrophages and dendritic cells was involved in the pathogenesis of mouse colitis (33, 43). Furthermore, pectin and galactan, the oligosaccharide component of side chains, were reported to directly interact with TLR2 and 4 and to block downstream signaling of TLR2/4 (17, 44). These studies prompted us to examine whether pectin directly regulates activation of TLR2 and TLR4 in myeloid cells. To investigate this possibility *in vitro*, we treated cultures of the macrophage cell line RAW264.7 with citrus and orange pectin followed by stimulation with Pam3CSK4 or LPS. Consistent with the *in vivo* results, we found a marked reduction in IL-6 production under stimulation with Pam3CSK4 or LPS in cells pre-treated with orange pectin but not citrus pectin (Figures 6A,B). To assess the anti-inflammatory activity of side chain, we treated cells with polysaccharides derived from side chains of orange and citrus pectin. Treatment of side chain polysaccharides significantly suppressed IL-6 production in LPS-stimulated RAW264.7 cells and the inhibitory effect on IL-6 production was comparable between the orange and citrus polysaccharides (Figure 6C). These results suggested that both side chain of citrus and orange pectin contained polysaccharides that directly interact with the macrophages and regulate the production of at least one inflammatory cytokine.

### Orange Pectin, Not Citrus Pectin, Ameliorates DSS-Induced Colitis

**Figure 7:**
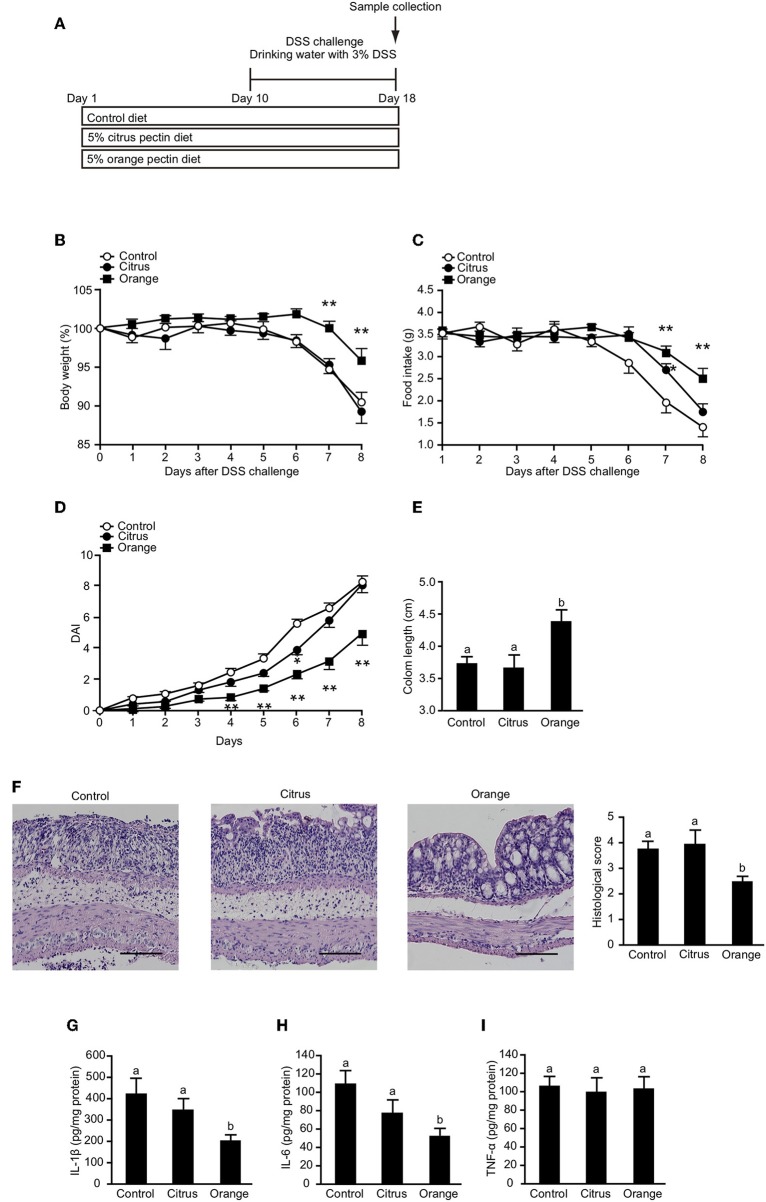
Effects of pectin feeding on dextran sulfate sodium (DSS)-induced colitis in mice. **(A)** Experimental design and time-course of treatment. Changes in **(B)** body weight and **(C)** food intake, and **(D)** disease activity index (DAI) score for the 8 days following DSS administration. Colon length **(E,F)** representative histological section images and score 8 days following DSS administration. Levels of colonic **(G)** interleukin (IL)-1β, **(H)** IL-6, and **(I)** tumor necrosis factor (TNF)-α 8 days following DSS administration. Scale bars: 100 μm. Values are presented as means ± standard error of the mean (*n* = 10–12). Values not sharing a common letter (a or b) were significantly different (*P* < 0.05). ^*^P < 0.05; ^**^P < 0.01.

To confirm that orange pectin affects myeloid cells including macrophages but not lymphocytes, we assessed the protective effect of orange pectin against DSS-induced colitis (Figure 7A), which does not require T or B lymphocytes (45). Control and citrus pectin-fed mice exhibited weight loss exceeding 10% of body weight (Figure 7B) and decreased daily food intake of approximately 2 g at day 8 following DSS administration (Figure 7C). Similar to the mice with TNBS-induced colitis, orange pectin-fed mice had milder weight loss and a diminished effect on the reduction in food intake compared with control mice (Figures 7B,C). In addition, only orange pectin feeding significantly improved the DAI score (Figure 7D) and shortening of colon length (Figure 7E). Furthermore, the histological score was also improved in orange pectin-fed mice (Figure 7F). Levels of colonic IL-1β and IL-6 in orange pectin-fed mice were significantly decreased compared with control and citrus pectin-fed mice (Figures 7G,H). However, the level of TNF-α in the colon was unchanged in orange pectin-fed mice (Figure 7I). Knowing that intestinal macrophages have been reported to supply IL-6 and raise the inflammatory response in DSS-induced colitis (46), these results indicated that orange pectin regulates IL-6 production in colon, thereby attenuating the inflammatory response.

## Discussion

In the present study, we found that orange pectin, but not citrus pectin, exerted a protective effect against TNBS- and DSS-induced colitis (Figures 1, 7). Because orange pectin has a higher number of neutral sugar side chains than citrus pectin, it was unsurprising to find that the protective effect of pectin against colitis was mediated in a side chain-dependent manner. This is the first study to indicate the significance of side chains in the protection against colitis.

Orange pectin feeding temporary increased the fecal concentration of propionic acid (Figure 4B) but did not change the concentrations of acetic acid and butyric acid (Figures 4A,C). We surmise that orange pectin feeding influences the microbial composition to promote propionic acid production. The genus *Bacteroides* is part of the indigenous microbiota of human and animal gastrointestinal tracts and is able to selectively generate propionic acid from succinate (47). In addition, some species of *Bacteroides* are known to contain more than twice the number of glycosidase and lyase genes than the human genome, which digest and utilize almost every polysaccharide of the major plants, including pectin (48). *In vitro* fermentation using human fecal samples demonstrated that arabinan derived from sugar beet pectin augmented the growth of *Bacteroides* and promoted the generation of propionic acid (49). Tian et al. also demonstrated that soy pectin, which contains more neutral sugar residues, promotes the generation of propionic acid and strongly stimulates the growth of *Bacteroides* better than citrus pectin (20). Therefore, the side chain content of pectin seems to increase the fecal concentration of propionic acid by expanding the *Bacteroides* population in the microbiota.

TNBS-induced colitis is believed to induce a T cell-mediated response against haptenized autologous antigen (23). However, orange pectin feeding did not regulate Treg differentiation (Figure 2C) or alter the production of butyric acid (Figure 4C), which is known to augment Treg differentiation (11). On the other hand, both orange and citrus pectin feeding suppressed the accumulation of colonic Th17 cells (Figure 2A). A massive amount of reactive oxygen species (ROS) is known to be generated in TNBS-induced colitis (50, 51), and ROS-mediated signaling is reported to give rise to enhanced Th17 differentiation (52). Because pectin-derived oligosaccharides reduce ROS generation by affecting the redox system (53), orange pectin and citrus pectin might be expected to suppress Th17 accumulation by regulating ROS generation. However, the suppression of Th17 by pectin seems to be insufficient to attenuate colitis symptoms because citrus pectin-fed mice exhibited disease severity that was comparable to control mice (Figure 1). We found that the number of colonic Th1 cells increased in orange pectin-fed mice (Figure 2B). Consistent with this result, propionic acid is reported to be a principal modulator for Th1 differentiation (54). Although the physiological significance of the increase in Th1 by SCFAs is unknown, Park et al. reported that the increase in Th1 population did not inhibit the anti-inflammatory effect of SCFAs. As it has been demonstrated that Th1 can produce not only IFN-γ but also anti-inflammatory cytokines (55), detailed analysis for cytokine profile are required to determine whether pectin-induced Th1 contribute to the pathogenesis of colitis.

Because bacterial proteins as well as colonic autologous antigens induce both innate and adaptive immune responses in the TNBS-induced colitis model (39), we hypothesized that orange pectin would also affect innate immune cells and regulate cell activation. A recent report showed that intestinal macrophage subsets are involved in the initiation of murine colitis (34). Furthermore, Kayama et al. reported that CX3CR1^+^ intestinal macrophages were able to produce high concentrations of IL-1β and IL-6 via activation of TLR4, and that suppression of this pathway resulted in the amelioration of DSS-induced colitis (46). Our present results showed that colonic IL-6 levels were decreased in orange pectin-fed mice both in TNBS- and DSS-induced colitis (Figures 5E, 7H). IL-6 has been shown to directly affect the infiltration of neutrophils and monocytes into the DSS-treated colon (56), and Lee et al. demonstrated that intestinal epithelial cells stimulated with IL-6 recruited lymphocytes and neutrophils to the intestinal mucosa by producing the proinflammatory protein S100A9 (57). Collectively, these results provide evidence that orange pectin ameliorates colitis at least in part by attenuating IL-6 production in colon.

In this study, we showed that the protective effect of orange pectin against TNBS-induced colitis was observed even in antibiotic-treated mice (Figure 5). We previously reported that pectin attenuates LPS-induced IL-6 production in macrophages in a side chain-dependent manner (16). Previous studies reported that neutralization of IL-6 improves DSS-induced colitis (40, 41), thus we assumed that orange pectin exerted stronger inhibitory effect on IL-6 production in macrophages than citrus pectin. Consistent with this assumption, orange pectin, not citrus pectin, significantly reduced IL-6 production in TLR2/4-stimulated cells (Figures 6A,B). It has been reported that not only pectin but also galactan and arabinogalactan interact with TLR2/4 (44, 58). Therefore, it is likely that the galactose and arabinose residues in orange pectin are important for its anti-inflammatory activity. However, the detailed structure of the carbohydrate responsible for this inhibitory activity has not been clarified because the chemical structures of galactan and arabinogalactan differ among plant species, and are known to have a wide variety. In addition, these *in vitro* results were correlated with colonic IL-6 levels in DSS colitis (Figure 7H) because DSS is thought to disrupt the integrity of the mucosal barrier, which allows commensal bacteria to elicit TLR2 and TLR4 dependent inflammation (59, 60). However, to date it remains unclear whether pectin directly interacts with colonic macrophages and regulates IL-6 production via its side chain. Future studies are required to determine whether intestinal macrophages directly sensitized by pectin contribute to the amelioration of colitis.

In conclusion, our present study suggests that pectin attenuates colitis at least in part via its side chain. Taken together with the well-established prebiotic effect of pectin in the protection against colitis (14, 15), we propose that pectin exhibits its anti-inflammatory effects in two ways: microbiota-dependent and microbiota-independent. These findings shed light on the novel mechanism by which pectin protects against colitis and suggest that pectin may be a promising prophylactic agent in the fight against IBD. Further analysis will be necessary to elucidate the detailed structure of the neutral sugar side chain required for protection against colitis.

## Data Availability Statement

All datasets generated for this study are included in the article/Supplementary Material.

## Ethics Statement

The experimental design of the animal study has been reviewed and approved by the Animal Research Committee of Gifu University, and animal care and use were in full compliance with the institutional guidelines of Gifu University.

## Author Contributions

KI, TY, and KK designed the study and wrote the paper. KI performed all of the experiments. TM performed the DSS-colitis experiments. All authors have read and approved the paper.

### Conflict of Interest

The authors declare that the research was conducted in the absence of any commercial or financial relationships that could be construed as a potential conflict of interest.

## Supplementary Material

Supplementary Figure 1 Schematic representation of pectin structure in citrus and orange.

Supplementary Figure 2 Effect of pectin feeding on the production of three intestinal short-chain fatty acids in mice pre-treated with antibiotics (Abx). Fecal samples were collected at 14 days after pectin feeding and used to determine the concentrations of **(A)** acetic acid, **(B)** propionic acid, and **(C)** butyric acid. Values are presented as means ± standard error of the mean (*n* = 5).

Supplementary Figure 3 Effect of antibiotic (Abx) treatment on genomic copy number of bacterial 16S rRNA in stools. DNA was extracted from fecal samples collected at 14 days after pectin feeding and 16S rRNA gene was quantified by quantitative PCR with bacterial universal primers. Values are presented as means ± SEM (*n* = 5). a–c, Values not sharing a common letter are significantly different (*p* < 0.05).
