# Supplementary material for: Dietary Fiber Pectin Ameliorates Experimental Colitis in a Neutral Sugar Side Chain-Dependent Manner
Source: Front Immunol. 2019 Dec 19;10:2979. doi: 10.3389/fimmu.2019.02979 (PMC6930924; doi:10.3389/fimmu.2019.02979)
Supplement: Supplementary file 1 [file Data_Sheet_1.docx]

**Supplementary information**

Fecal DNA extraction and quantitative PCR

Fecal DNA was extracted according to a method described by Matsuki *et al* [1]. with slight modifications. Twenty mg of stools was suspended in 980 µL MilliQ water and 200 µL of the suspension was mixed with extraction buffer (200 mM Tris-HCl, 80 mM EDTA, pH 9.0), 50 µL of 10% sodium dodecyl sulfate, 0.3 g of glass beads (φ 0.1 mm), and 500 µL of buffer-saturated phenol (Nacalai tesque, Kyoto, Japan). The mixture was homogenized at 2500 rpm for 30 sec using a multi-beads shocker (MB455GU(S): Yasui kikai, Osaka, Japan). After centrifugation, 250 µL of the supernatant was collected and purified by using spin column (Genedesign, Osaka, Japan). DNA extract was eluted to 10 µL in MilliQ water and was used in quantitative PCR. Relative copy number of 16S RNA gene per stool weight was determined by quantitative PCR with THUNDERBIRD SYBR qPCR Mix (Toyobo, Osaka, Japan) on a Thermal Cycler Dice Real Time System (Takara Bio, Shiga, Japan). The primer sequences used for quantitative PCR were as follows: 16S rRNA gene, 5'-TCCTACGGGAGGCAGCAGT-3' and 5'-GGACTACCAGGGTATCTAATCCTGTT-3'.

1. Matsuki T, Watanabe K, Fujimoto J, Kado Y, Takada T, Matsumoto K, Tanaka R. Quantitative PCR with 16S rRNA-gene-targeted species-specific primers for analysis of human intestinal bifidobacteria. *Appl Environ Microbiol*.(2004) **70**:167–73. doi:10.1128/aem.70.1.167-173.2004
