# Supplementary figures and images for: Dietary Fiber Pectin Ameliorates Experimental Colitis in a Neutral Sugar Side Chain-Dependent Manner
Source: Front Immunol. 2019 Dec 19;10:2979. doi: 10.3389/fimmu.2019.02979 (PMC6930924; doi:10.3389/fimmu.2019.02979)

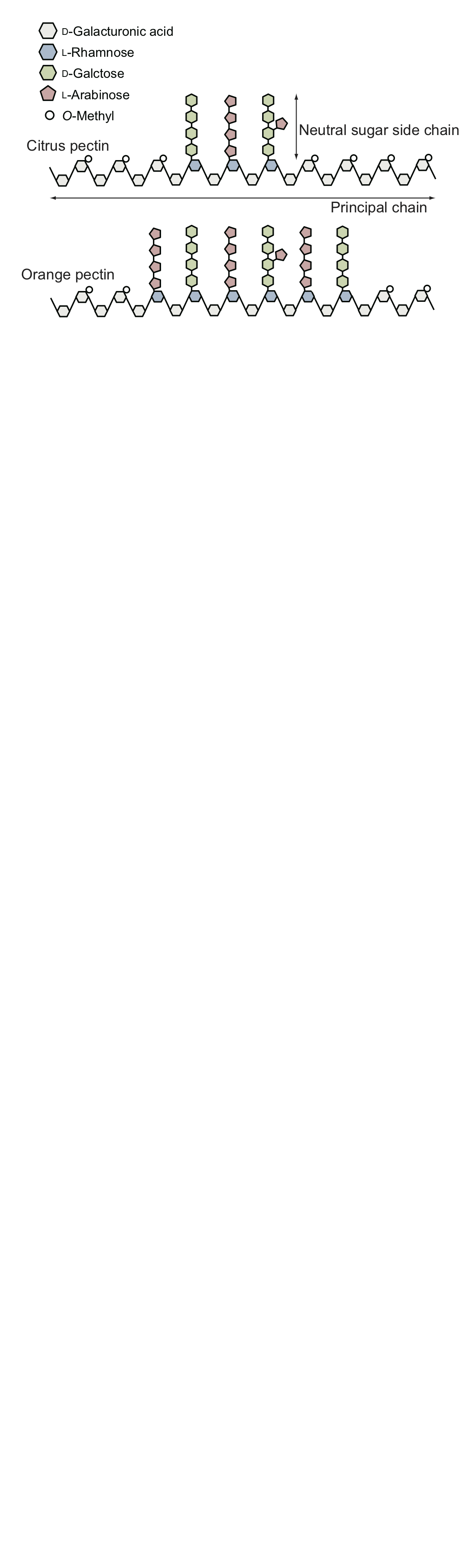

Supplement: Supplementary Figure 1 — Schematic representation of pectin structure in citrus and orange. [file Image_1.TIFF]

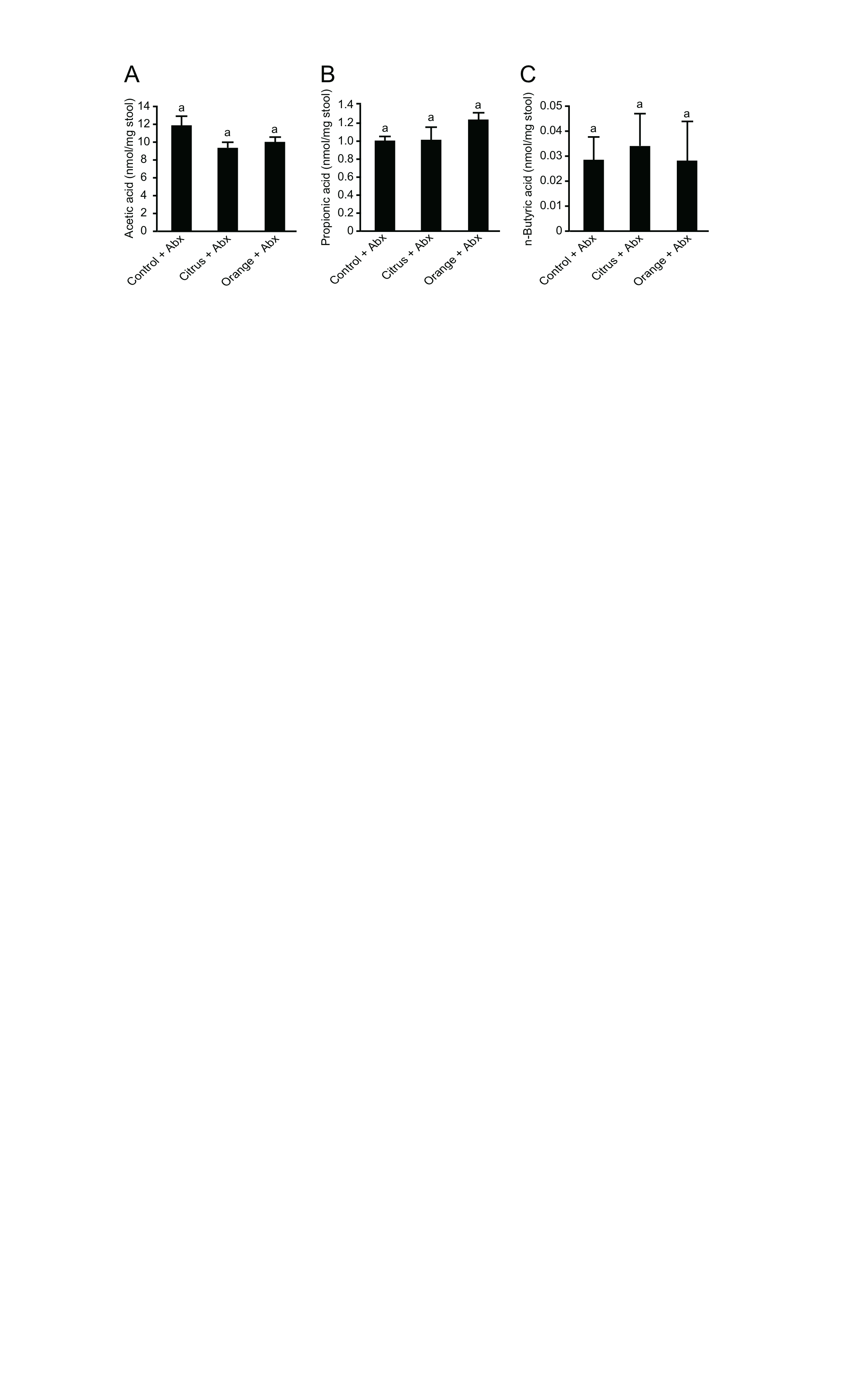

Supplement: Supplementary Figure 2 — Effect of pectin feeding on the production of three intestinal short-chain fatty acids in mice pre-treated with antibiotics (Abx). Fecal samples were collected at 14 days after pectin feeding and used to determine the concentrations of (A) acetic acid, (B) propionic acid, and (C) butyric acid. Values are presented as means ± standard error of the mean (n = 5). [file Image_2.TIFF]

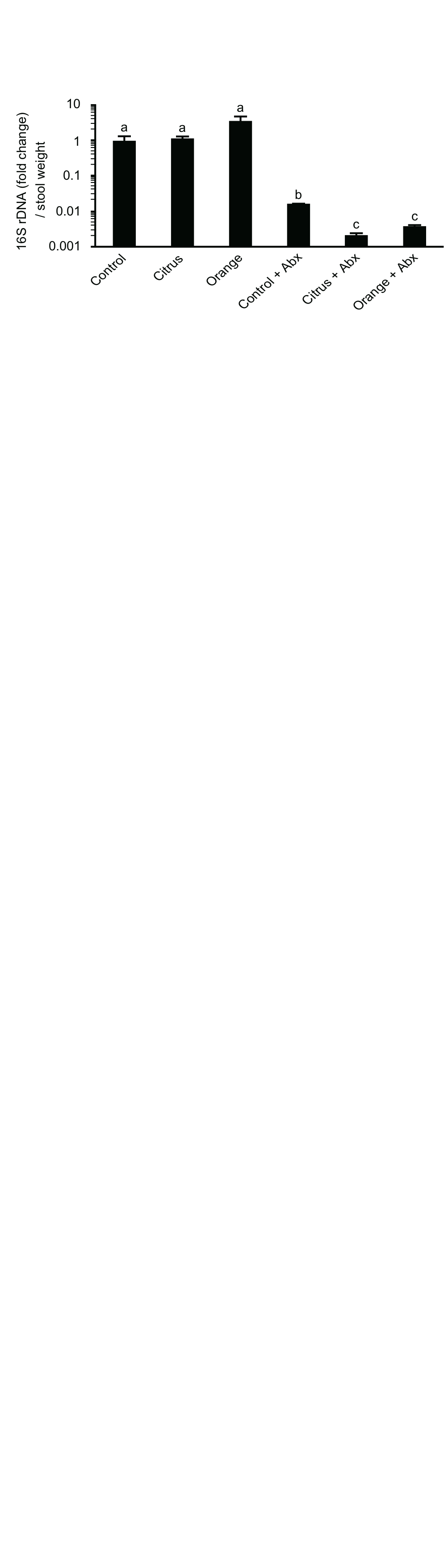

Supplement: Supplementary Figure 3 — Effect of antibiotic (Abx) treatment on genomic copy number of bacterial 16S rRNA in stools. DNA was extracted from fecal samples collected at 14 days after pectin feeding and 16S rRNA gene was quantified by quantitative PCR with bacterial universal primers. Values are presented as means ± SEM (n = 5). a–c, Values not sharing a common letter are significantly different (p < 0.05). [file Image_3.TIFF]
